# Factors associated with 36-month loss to follow-up and mortality outcomes among HIV-infected adults on antiretroviral therapy in Central Kenya

**DOI:** 10.1186/s12889-020-8426-1

**Published:** 2020-03-14

**Authors:** Paul Wekesa, Angela McLigeyo, Kevin Owuor, Jonathan Mwangi, Evelyne Nganga, Kenneth Masamaro

**Affiliations:** 1 Centre for Health Solutions, Nairobi, Kenya; 2 Division of Global HIV & TB, Centers for Disease Control and Prevention (CDC), Nairobi, Kenya

**Keywords:** Antiretroviral therapy, HIV, Kenya, Loss to follow-up, Mortality

## Abstract

**Background:**

The scale-up of HIV treatment programs has resulted in a reduction in HIV-related morbidity and mortality. However, retention of patients in these programs remains a challenge in sub-Saharan Africa. Understanding factors associated with loss to follow-up (LTFU) and mortality outcomes is therefore important to inform targeted program interventions.

**Methods:**

A retrospective multi-cohort analysis of 23,890 adult patients on ART over 36 months of follow-up in Kenya was done. Multivariate logistic regression analysis was done to assess for factors associated with LTFU and mortality at 6, 12, 24, and 36 months of follow-up.

**Results:**

Majority, 67.7%, were female. At 36 months, 27.2% were LTFU and 13.5% had died. Factors associated with mortality at 36 months included older age (51 years and above) using 20–35 years as reference [(adjusted odds ratio [aOR], 1.51, 95% confidence interval (CI) 1.23–1.86, *p* < 0.001], being male (aOR, 1.59, 95% CI 1.39–1.83, *p* < 0.001), divorced using married as reference (aOR, 1.86, 95% CI 1.56–2.22, *p* < 0.001), having a body mass index (BMI) score of less than 18.5 kg/m^2^ using 18.5–24.9 kg/m^2^ as reference (aOR = 1.79, 95% CI 1.52–2.11, *p* < 0.001), and, World Health Organization stage III and IV using stage I as the reference (aOR, 1.94, 95% CI 1.43–2.63 and aOR, 4.24, 95% CI 3.06–5.87, *p* < 0.001 respectively). Factors associated with LTFU at 36 months included being young between 20 and 35 years (aOR, 1.49, 95% CI 1.40–1.59, *p* < 0.001) using 36–50 years as reference, being male (aOR, 1.19, 95% CI 1.12–1.27, *p* < 0.001), and being single or divorced using married as reference (aOR, 1.34, 95% CI 1.23–1.45 and aOR, 1.25, 95% CI 1.15–1.36, *p* < 0.001 respectively). Patients with baseline BMI of less than 18.5 kg/m^2^ using normal BMI as reference (aOR, 1.68, 95% CI 1.39–2.02, *p* < 0.001) were also likely to be LTFU.

**Conclusions:**

Factors associated with LTFU and mortality were generally similar over time. Implementation of programs in similar settings should be tailored to gender, age profiles, nutritional, and, marital status of patients to address LTFU. In addition, programs should focus on the care of older patients to reduce the risk of mortality.

## Background

Access to antiretroviral therapy (ART) has been scaled up globally, reaching 21.7 million individuals by 2017. These efforts have contributed to a decline in AIDS-related deaths by 42% from 2007 to 2017 in eastern and southern Africa [1]. HIV programs in Kenya have grown considerably in the past decade due to rapid scale-up of treatment programs. In 2017, the prevalence of HIV among adults aged 15–49 in Kenya was estimated at 4.9% [2]. Out of the estimated 1.4 million adults aged 15 years and above living with HIV, approximately 1,035,615 (75%) were on ART by 2017 [2].

Kenya has had six iterations of the national HIV treatment guidelines since 2004, to ensure HIV-infected patients receive quality care [3, 4]. The expansion of HIV services has resulted in a reduction in HIV-related morbidity and mortality in the country [2]. HIV programs, however, continue to report an increase in both mortality and loss to follow-up (LTFU) outcomes with retention being less than optimal [5]. Retention on ART remains a challenge for HIV treatment programs in sub-Saharan Africa (SSA). After 36-months of follow-up, only 65% of those on ART in Africa were retained on treatment in a meta-analysis of studies conducted in 42 low- and middle income countries [6]. Also, LTFU through undocumented transfers and consequent treatment interruptions increased with each year of follow-up for patients traced after LTFU in a meta-analysis that included 12 countries in SSA [7]. Understanding the factors associated with treatment outcomes in various contexts is important for developing targeted interventions to improve patient retention.

Factors associated with treatment outcomes have been investigated in SSA countries. A study in Nigeria reported that being on ART, age above 30 years, being female, diagnosis at WHO clinical stage I and II, CD4 count greater than 100 cells/μ, longer duration on ART, and non-stavudine-based regimens were associated with good retention [8]. Another study at primary care clinics in South Africa reported a weak association between good retention and psychosocial support to the patient [9].

Several other studies have reported predictors of LTFU including younger or older age, male gender, low baseline CD4, World Health Organization (WHO) stage IV, low baseline body weight, greater than 10% body weight loss, low body mass index scores (BMI), and seeking care from higher level healthcare facilities [10–15], while predictors of mortality included young age between 15 and 24 years, or older age above 50 years, male gender, low baseline CD4 counts, and clinical AIDS prior to ART initiation [16–19].

In Kenya, studies have been done to evaluate factors associated with LTFU and mortality outcomes among patients on ART. However, many of these studies have investigated outcomes for less than 36-month cohorts. In addition, these studies did not investigate temporal changes in these outcomes. This study aimed to evaluate factors that affect LTFU and mortality outcomes including temporal changes for 36-month patient cohorts enrolled on ART between 2004 and 2015.

## Methods

### Study setting and period

Centre for Health Solutions - Kenya is a not-for-profit organization that supported HIV care and treatment services in 5 counties in Central Kenya through the Tegemeza Project, with funding from the President’s Emergency Plan for AIDS Relief (PEPFAR). The counties were Kiambu (estimated population of 1,831,800, 68,349 adult PLHIV and adult HIV prevalence of 5.6% in 2015), Murang’a (estimated population of 1,063,721, 26,238 adult PLHIV and adult HIV prevalence of 4.2% in 2015), Nyeri (estimated population of 782,864, 17,973 adult PLHIV and adult HIV prevalence of 3.4% in 2015), Nyandarua (estimated population of 673,000, 12,283 adult PLHIV and adult HIV prevalence of 3.0% in 2015) and Laikipia (estimated population of 487,934, 7036 adult PLHIV and adult HIV prevalence of 3.2% in 2015) as per Kenya HIV estimates report, 2015 [20]. The HIV prevalence rates in 2017 were reported as 4.0% in Kiambu, 4.2% in Muranga, 3.7% in Nyeri, 3.5% in Nyandarua, and 2.7% in Laikipia [2]. HIV care and treatment services have been decentralized to county hospitals and health centers to increase the number of people living with HIV (PLHIV) accessing health services. Services were provided in comprehensive care clinics through a multi-disciplinary team composed of clinical officers, nurses, data clerks, peer educators, nutritionists, social workers, pharmacists, pharmaceutical technologists, and laboratory technologists. All health facilities offered a standard package of care that consisted of provider initiated testing and counselling (PITC), same day enrolment into the HIV clinic, patient education and counselling, baseline CD4 count testing, ART initiation for eligible patients, treatment monitoring, structured follow-up appointments as per national guidelines, comorbidity management, as well as adherence and retention support. All HIV services were offered in an integrated model that aimed to combine all services required by the patient in a one-stop-shop approach.

### Study design, population, and sample

This was a retrospective cohort analysis of adults on ART. The analysis targeted all adults aged 20 years and above initiating ART between 2004 and 2012, with a 36-month follow-up, in all 104 (101 government owned and 3 private owned) ART and prevention of mother-to-child transmission of HIV (PMTCT) clinics in the 5 counties. The number of study facilities per county were 36 in Nyeri, 33 in Muranga, 26 in Kiambu, 7 in Nyandarua, and 2 in Laikipia. The number of clients included in the analysis was 23,890.

### Data sources

The data were extracted from the Comprehensive Patient Application Database and International Quality Care (IQare) electronic medical record (EMR) systems. Manual data were entered into the EMR systems by data clerks at health facilities and contained longitudinal data updated at every clinic visit from the national patient-level data recording tool, the Ministry of Health, Comprehensive Care Clinic Patient Card (MOH 257).

### Variables and definitions

Variables included in this analysis were age, gender, marital status, baseline CD4 count, baseline WHO stage, baseline BMI, ART regimen at initiation (composed of a backbone of stavudine [D4T], zidovudine [AZT], abacavir [ABC] or tenofovir [TDF]). The outcomes of interest were LTFU and mortality at 6, 12, 24, and 36 months of follow up as per PEPFAR and Kenya national ART outcome monitoring guidelines. A client was considered LTFU if he/she missed a clinic appointment for more than 90 days consecutively per PEPFAR and Kenya national ART outcome monitoring guidelines.

### Ethics

Ethical approvals were obtained prior to initiating the study. Local ethical approval was obtained from the Kenyatta National Hospital and University of Nairobi Institutional Review Boards (P339/06/2013). The protocol was also reviewed in accordance with the Centers for Disease Control and Prevention (CDC) human research protection procedures and was determined to be research, but CDC investigators did not interact with human subjects or have access to identifiable data or specimens for research purposes. Anonymity and confidentiality of data was strictly maintained.

### Data analysis

Descriptive data analysis was done presenting counts and respective percentages for all the categorical variables such as gender and marital status. Median and corresponding interquartile range (IQR) was presented for the age variable. Separate univariate and multivariate logistic regression analyses were done for each time point to assess for factors associated with LTFU and mortality at 6, 12, 24, and 36 months.

Routinely collected data at health facility level were used. From the assessment, four out of seven variables had missing data including baseline WHO stage (23.04%), baseline BMI (46.14%), marital status (7.36%) and baseline CD4 (19.5%) count. We assumed the data was missing at random and therefore missing values could be fully accounted for by complete measured variables. To deal with missing data, all the regression models were estimated after multiple imputations of missing values using chained eqs [20].. A generally sufficient number of ten imputed datasets were used to calculate mean estimates of the variables of interest using Rubin’s rules [20]. We also performed diagnostic plots assessing the observed and imputed values to determine if the imputations were reasonable.

The multivariate models adjusted for patients’ cohort year, demographic and clinical characteristics which were included a priori. Crude Odds Ratios (OR) and corresponding 95% confidence intervals (CIs) were presented for the univariate analysis. The adjusted Odds Ratios (aOR) from multivariate analysis for the odds of LTFU and mortality at each time point were also presented. All statistical tests were evaluated at the 5% level of significance. All the analyses were done in Stata version 15.1 (StataCorp. 2017. *Stata Statistical Software: Release 15*. College Station, TX: StataCorp LLC.)

## Results

### Patients’ characteristics at ART initiation

The analysis included 23,890 patients, who initiated ART between January 1, 2004 and December 31, 2012 and completed 36 months of follow-up by March 30, 2015. The majority, 16,182 (67.7%), were female and slightly less than half 10,827 (45.3%), were between ages 20 to 35 years. About 47.0% (11,143) were married. Median age at ART initiation was 38 years (IQR: 37–44), 80.6% had CD4 cell counts less than 250 cells/ *μ*L and only 0.7% had CD4 cell counts greater than 500 cells/μL. Forty-five percent of patients had WHO clinical stage III and IV disease and 26.9% had a BMI below 18.5 kg/m^2^. More than half (54.2%) were initiated on a D4T-based ART regimen.

### Temporal changes in patient baseline characteristics and outcomes

The number of patients initiating ART increased each calendar year from 368 in 2004 to 2968 in 2012. Median baseline CD4 count increased in the period from 96 cells cells/ *μ*L (IQR: 38–170) in 2004 to 197 cells cells/μL (IQR: 85–286) in 2012. The proportion of patients with WHO clinical stage III and IV disease decreased from 30.5% (101) in 2004 to 2.9% (76) in 2012.

After 36 months of follow up, a total of 3222 patients (13.5%) had died, 556 (2.3%) within first 6 months and 1027 (4.3%) between 25 and 36 months on ART. With regard to LTFU, 2451 (10.3%) of patients were LTFU at 6 months and 6499 (27.2%) between 25 and 36 months. The descriptive information of study participants in the cohort, including mortality and LTFU outcomes at 6, 12, 24, and 36 months of ART are presented in Table 1. The overall retention was 87.4% at 6 months and 68.5% at 36 months of follow up.

**Table 1:** Descriptive characteristics of HIV-infected adults on antiretroviral therapy by mortality and LTFU outcomes

	Mortality				LTFU			
	6 months	12 months	24 months	36 months	6 months	12 months	24 months	36 months
	n (%)	n (%)	n (%)	n (%)	n (%)	n (%)	n (%)	n (%)
Gender								
Female (*n* = 16182)	304 (54.7)	395 (55.2)	519 (56.2)	576 (56.1)	1549 (63.2)	2272 (64.7)	3358 (65.6)	4282 (65.9)
Male (*n* = 7708)	252 (45.3)	320 (44.8)	405 (43.8)	451 (43.9)	902 (36.8)	1241 (35.3)	1759 (34.4)	2217 (34.1)
Total (*n* = 23890)								
Age								
20–35 (*n* = 10827)	229 (41.2)	281 (39.3)	370 (40.0)	414 (40.3)	1203 (49.1)	1797 (51.2)	2675 (52.3)	3369 (51.8)
36–50 (*n* = 10629)	255 (45.9)	336 (47.0)	425 (46.0)	470 (45.8)	1016 (41.5)	1404 (40.0)	2001 (39.1)	2558 (39.4)
51+ (*n* = 2434)	72 (12.9)	98 (13.7)	129 (14.0)	143 (13.9)	232 (9.5)	312 (8.9)	441 (8.6)	572 (8.8)
Total (*n* = 23890)								
Marital Status								
Divorced (*n* = 3923)	91 (17.7)	126 (19.0)	165 (19.1)	178 (18.8)	451 (21.0)	644 (20.6)	906 (19.7)	1193 (20.3)
Married (*n* = 11143)	203 (39.6)	273 (41.1)	353 (41.0)	385 (40.7)	963 (44.7)	1422 (45.5)	2128 (46.4)	2738 (46.7)
Single (*n* = 4091)	146 (28.5)	179 (27.0)	224 (26.0)	251 (26.5)	487 (22.6)	699 (22.4)	1014 (22.1)	1235 (21.1)
Widowed (*n* = 2975)	73 (14.2)	86 (13.0)	120 (13.9)	133 (14.0)	251 (11.7)	359 (11.5)	541 (11.8)	698 (11.9)
Total (*n* = 22132)								
BMI								
< 18.5 (*n* = 3466)	123 (49.2)	148 (47.6)	191 (46.8)	210 (47.1)	326 (40.3)	441 (36.7)	633 (33.0)	819 (30.9)
18.5–24.9 (*n* = 6887)	110 (44.0)	137 (44.1)	181 (44.4)	197 (44.2)	399 (49.4)	623 (51.9)	1027 (53.6)	1437 (54.3)
25–29.9 (*n* = 1708)	13 (5.2)	19 (6.1)	26 (6.4)	29 (6.5)	59 (7.3)	96 (8)	178 (9.3)	264 (10.0)
30+ (*n* = 807)	4 (1.6)	7 (2.3)	10 (2.5)	10 (2.2)	24 (3.0)	41 (3.4)	79 (4.1)	128 (4.8)
Total (*n* = 12868)								
Baseline CD4								
0–250 (*n* = 15483)	387 (91.7)	491 (90.9)	648 (91.7)	715 (90.9)	1615 (86.5)	2343 (85.9)	3357 (83.5)	4275 (83.1)
251–500 (*n* = 3611)	34 (8.1)	48 (8.9)	56 (7.9)	69 (8.8)	236 (12.6)	366 (13.4)	630 (15.7)	830 (16.1)
Above 500 (*n* = 124)	1 (0.2)	1 (0.2)	3 (0.4)	3 (0.4)	15 (0.8)	19 (0.7)	33 (0.8)	39 (0.8)
Total (*n* = 19218)								
Regimen started								
ABC-based (*n* = 370)	8 (1.4)	8 (1.1)	9 (1.0)	9 (0.9)	82 (3.3)	113 (3.2)	148 (2.9)	166 (2.6)
AZT-based (*n* = 4465)	102 (18.3)	130 (18.2)	156 (16.9)	167 (16.3)	371 (15.1)	544 (15.5)	827 (16.2)	1102 (17.0)
D4T-based (*n* = 12949)	360 (64.7)	466 (65.2)	613 (66.3)	690 (67.2)	1556 (63.5)	2178 (62.0)	3062 (59.8)	3728 (57.4)
TDF-based (*n* = 6106)	86 (15.5)	111 (15.5)	146 (15.8)	161 (15.7)	442 (18.0)	678 (19.3)	1080 (21.1)	1503 (23.1)
Total (*n* = 23890)								
WHO clinical stage at ART initiation								
Stage I (*n* = 3310)	30 (6.4)	40 (6.7)	53 (6.9)	63 (7.5)	231 (13.5)	354 (14.2)	572 (15.5)	763 (16.1)
Stage II (*n* = 6800)	104 (22.3)	136 (22.6)	183 (23.8)	208 (24.7)	535 (31.4)	832 (33.4)	1267 (34.3)	1661 (34.9)
Stage III (*n* = 7282)	249 (53.3)	322 (53.6)	404 (52.5)	436 (51.8)	801 (47.0)	1132 (45.5)	1632 (44.1)	2047 (43.1)
Stage IV (*n* = 994)	84 (18.0)	103 (17.1)	129 (16.8)	135 (16.0)	138 (8.1)	171 (6.9)	226 (6.1)	282 (5.9)
Total (*n* = 18386)								
Dead	556 (2.3)	715 (3.0)	924 (3.9)	1027 (4.3)				
LTFU					2451 (10.3)	3513 (14.7)	5117 (21.4)	6499 (27.2)

### Factors associated with mortality in multivariate analysis

Mortality was associated with male gender (6 month aOR, 1.66, 95% CI 1.38–2.00, *p* < 0.001 and 36 month aOR, 1.59, 95% CI 1.39–1.83, *p* < 0.001), older age 51 years and above using 20–35 years as reference (6 month aOR, 1.34, 95% CI 1.00–1.78, *p* = 0.047 and 36 month aOR, 1.51, 95% CI 1.23–1.86, *p* < 0.001), and, being single (6 month aOR, 1.30, 95% CI 1.00–1.68, *p* = 0.050 and 36 month aOR, 1.36, 95% CI 1.13–1.63, *p* = 0.001) or divorced (6 month aOR, 1.99, 95% CI 1.58–2.51, *p* < 0.001 and 36 month aOR, 1.86, 95% CI 1.56–2.22, *p* < 0.001) using married as reference. Mortality was also associated with advanced WHO clinical stage III, using stage I as the reference, (6 month aOR, 2.27, 95% CI 1.49–3.47, *p* < 0.001 and 36 month aOR, 1.94, 95% CI 1.43–2.63, *p* < 0.001) and clinical stage IV (6 month aOR, 5.29, 95% CI 3.45–8.10, *p* < 0.001 and 36 month aOR, 4.24, 95% CI 3.06–5.87, *p* < 0.001), and low BMI of less than 18.5 kg/m^2^ using 18.5–24.9 kg/m^2^ as reference (6 month aOR, 1.65, 95% CI 1.32–2.06, *p* < 0.001 and 36 month aOR, 1.54, 95% CI 1.31–1.82, *p* < 0.001). Patients initiated on a TDF-based ART regimen had lower odds of mortality using a D4T-based regimen as reference (6 month aOR, 0.58, 95% CI 0.46–0.74, *p* < 0.001 and 36 month aOR, 0.55, 95% CI 0.46–0.65, *p* < 0.001). Among those initiated on an AZT-based ART regime, mortality risk was lower later in treatment using D4T-based regimen as reference (36 month aOR 0.83, 95% CI 0.70–0.99, *p* = 0.040). Univariate and multivariate analysis of these factors are presented in Tables 2 and 3 respectively.

**Table 2:** Univariate analysis of factors associated with mortality among HIV-infected adults on antiretroviral therapy

	Univariable Results (*n* = 23890)							
	6 months		12 months		24 months		36 months	
	OR (95% CI)	*P* value	OR (95% CI)	*P* value	OR (95% CI)	*P* value	OR (95% CI)	*P* value
Gender								
Male	1.77 (1.49–2.09)	< 0.001	1.73 (1.49–2.01)	< 0.001	1.67 (1.47–1.91)	< 0.001	1.68 (1.48–1.91)	< 0.001
Female	Ref		Ref		Ref		Ref	
Age groups								
20–35	Ref		Ref		Ref		Ref	
36–50	1.14 (0.95–1.36)	0.162	1.23 (1.04–1.44)	0.013	1.18 (1.02–1.36)	0.024	1.16 (1.02–1.33)	0.028
51+	1.41 (1.08–1.85)	0.012	1.57 (1.25–1.99)	< 0.001	1.58 (1.29–1.94)	< 0.001	1.57 (1.29–1.91)	< 0.001
Marital Status								
Single	1.3 (1.01–1.68)	0.043	1.35 (1.08–1.68)	0.008	1.36 (1.13–1.64)	0.002	1.34 (1.12–1.6)	< 0.001
Divorced	1.96 (1.58–2.43)	< 0.001	1.80 (1.49–2.18)	< 0.001	1.76 (1.49–2.08)	< 0.001	1.79 (1.53–2.11)	< 0.001
Married	Ref		Ref		Ref		Ref	
Widowed	1.34 (1.01–1.77)	0.043	1.19 (0.92–1.53)	0.186	1.29 (1.04–1.60)	0.023	1.3 (1.05–1.61)	0.014
BMI								
< 18.5	1.96 (1.56–2.47)	< 0.001	1.85 (1.52–2.26)	< 0.001	1.82 (1.53–2.17)	< 0.001	1.79 (1.52–2.11)	< 0.001
18.5–24.9	Ref		Ref		Ref		Ref	
25–29.9	0.50 (0.32–0.78)	0.002	0.54 (0.36–0.81)	0.003	0.57 (0.41–0.8)	0.001	1.10 (0.63–1.94)	0.001
30+	0.37 (0.17–0.81)	0.013	0.45 (0.24–0.85)	0.013	0.53 (0.31–0.91)	0.02	0.54 (0.34–0.88)	0.013
CD4								
0–250	2.35 (0.39–14.22)	0.354	2.23 (0.45–11.11)	0.329	1.42 (0.48–4.23)	0.528	1.52 (0.51–4.49)	0.453
251–500	0.91 (0.14–5.83)	0.923	0.94 (0.18–4.77)	0.938	0.57 (0.18–1.74)	0.320	0.66 (0.22–2.00)	0.460
Above 500	Ref		Ref		Ref		Ref	
Regimen Started								
D4T-based	Ref		Ref		Ref		Ref	
ABC-based	0.77 (0.38–1.57)	0.476	0.59 (0.29–1.2)	0.146	0.50 (0.26–0.98)	0.042	0.44 (0.23–0.86)	0.016
AZT-based	0.82 (0.65–1.02)	0.076	0.80 (0.66–0.98)	0.147	0.73 (0.61–0.87)	< 0.001	0.69 (0.58–0.82)	< 0.001
TDF-based	0.50 (0.39–0.63)	< 0.001	0.50 (0.40–0.61)	0.148	0.49 (0.41–0.59)	< 0.001	0.48 (0.40–0.57)	< 0.001
WHO Stage clinical stage at ART initiation								
Stage I	Ref		Ref		Ref		Ref	
Stage II	1.56 (1.03–2.37)	0.036	1.57 (1.07–2.30)	0.02	1.56 (1.11–2.18)	0.01	1.50 (1.09–2.06)	0.012
Stage III	3.37 (2.23–5.10)	< 0.001	3.32 (2.29–4.82)	< 0.001	3.10 (2.26–4.25)	< 0.001	2.84 (2.11–3.83)	< 0.001
Stage IV	8.18 (5.38–12.44)	< 0.001	7.68 (5.27–11.20)	< 0.001	7.25 (5.23–10.04)	< 0.001	6.41 (4.68–8.78)	< 0.001

**Table 3:** Multivariate analysis of factors associated with mortality among HIV-infected adults on antiretroviral therapy

	Multivariate Results (*n* = 23890)							
	6 months		12 months		24 months		36 months	
(*n* = 23890)	aOR (95% CI)	*P* value	aOR (95% CI)	*P* value	aOR (95% CI)	*P* value	aOR (95% CI)	*P* value
Gender								
Male	1.66 (1.38–2.00)	< 0.001	1.59 (1.35–1.87)	< 0.001	1.56 (1.35–1.80)	< 0.001	1.59 (1.39–1.83)	< 0.001
Female	Ref		Ref		Ref		Ref	
Age groups								
20–35	Ref		Ref		Ref		Ref	
36–50	1.06 (0.88–1.28)	0.545	1.15 (0.97–1.37)	0.098	1.10 (0.95–1.27)	0.223	1.09 (0.94–1.25)	0.248
51+	1.34 (1.00–1.78)	0.047	1.54 (1.2–1.98)	0.001	1.52 (1.22–1.90)	< 0.001	1.51 (1.23–1.86)	< 0.001
Marital Status								
Single	1.30 (1.00–1.68)	0.05	1.34 (1.07–1.68)	0.01	1.36 (1.12–1.65)	0.002	1.36 (1.13–1.63)	0.001
Divorced	1.99 (1.58–2.51)	< 0.001	1.86 (1.52–2.28)	< 0.001	1.80 (1.50–2.15)	< 0.001	1.86 (1.56–2.22)	< 0.001
Married	Ref		Ref		Ref		Ref	
Widowed	1.33 (0.99–1.78)	0.055	1.14 (0.88–1.49)	0.330	1.23 (0.98–1.55)	0.071	1.26 (1.01–1.58)	0.041
BMI								
< 18.5	1.65 (1.32–2.06)	< 0.001	1.57 (1.29–1.91)	< 0.001	1.55 (1.30–1.85)	< 0.001	1.54 (1.31–1.82)	< 0.001
18.5–24.9	Ref		Ref		Ref		Ref	
25–29.9	0.58 (0.37–0.9)	0.015	0.62 (0.41–0.94)	0.023	0.65 (0.46–0.92)	0.014	0.68 (0.50–0.92)	0.013
30+	0.41 (0.18–0.9)	0.026	0.49 (0.26–0.92)	0.027	0.57 (0.34–0.98)	0.04	0.58 (0.36–0.94)	0.027
CD4								
0–250	2.66 (0.46–15.43)	0.275	2.51 (0.53–12.00)	0.249	1.59 (0.56–4.53)	0.388	1.68 (0.59–4.77)	0.331
251–500	1.56 (0.26–9.47)	0.628	1.59 (0.33–7.76)	0.565	0.94 (0.32–2.78)	0.913	1.09 (0.37–3.18)	0.873
Above 500	Ref		Ref		Ref		Ref	
Regimen Started								
D4T-based	Ref		Ref		Ref		Ref	
ABC-based	0.95 (0.46–1.95)	0.889	0.71 (0.35–1.45)	0.346	0.60 (0.31–1.18)	0.140	0.52 (0.26–1.02)	0.056
AZT-based	1.02 (0.81–1.28)	0.896	0.99 (0.80–1.21)	0.887	0.89 (0.74–1.07)	0.214	0.83 (0.70–0.99)	0.04
TDF-based	0.58 (0.46–0.74)	< 0.001	0.57 (0.46–0.71)	< 0.001	0.57 (0.47–0.69)	< 0.001	0.55 (0.46–0.65)	< 0.001
Baseline WHO Stage								
Stage I	Ref		Ref		Ref		Ref	
Stage II	1.31 (0.86–2.01)	0.214	1.32 (0.90–1.95)	0.156	1.3 (0.93–1.83)	0.130	1.25 (0.91–1.72)	0.167
Stage III	2.27 (1.49–3.47)	< 0.001	2.29 (1.56–3.35)	< 0.001	2.11 (1.53–2.91)	< 0.001	1.94 (1.43–2.63)	< 0.001
Stage IV	5.29 (3.45–8.1)	< 0.001	5.10 (3.46–7.50)	< 0.001	4.79 (3.44–6.67)	< 0.001	4.24 (3.06–5.87)	< 0.001

### Factors associated with LTFU in multivariate analysis

LTFU was associated with male gender (6 month aOR, 1.23, 95% CI 1.12–1.36, *p* < 0.001 and 36 month aOR, 1.19, 95% CI 1.12–1.27, *p* < 0.001), younger age, 20 to 35 years using 36–50 years as reference (6 month aOR, 1.23, 95% CI 1.12–1.35, *p* < 0.001 and 36 month aOR, 1.49, 95% CI 1.40–1.59, *p* < 0.001) being single (6 month aOR, 1.34, 95% CI 1.19–1.51, *p* < 0.001 and 36 month aOR, 1.34, 95% CI 1.23–1.45, *p* < 0.001) and being divorced (6 month aOR, 1.35, 95% CI 1.19–1.53, *p* < 0.001 and 36 month aOR, 1.25, 95% CI 1.15–1.36, *p* < 0.001) using married as reference. Risk of LTFU was also higher, using WHO clinical stage I as the reference group, among those with advanced WHO clinical stage III (6 month aOR, 1.34, 95% CI 1.14–1.57, *p* < 0.001 and 36 month aOR, 1.22, 95% CI 1.12–1.34, *p* < 0.001) and stage IV (6 month aOR, 1.83, 95% CI 1.48–2.26, *p* < 0.001 and 36 month aOR, 1.34, 95% CI 1.15–1.57, *p* < 0.001), lower CD4 counts (6 month aOR, 1.34, 95% CI 1.16–1.55, *p* < 0.001 and 36 month aOR, 1.18, 95% CI 1.08–1.29, *p* < 0.001 for CD4 counts <=250 cells/μl) and low BMI < 18.5 kg/m^2^ compared to normal BMI (6 month aOR, 1.37, 95% CI 1.21–1.56, *p* < 0.001 and 36 month aOR, 1.16, 95% CI 1.06–1.27, *p* < 0.001). Patients on an ABC-based ART regimen were also significantly more likely to be LTFU using D4T-based regimen as reference (6 month aOR, 2.37, 95% CI 1.82–3.08, *p* < 0.001 and 36 month aOR, 2.17, 95% CI 1.75–2.69, *p* < 0.001). Univariate and multivariate regression analysis of these factors are presented in Tables 4 and 5 respectively.

**Table 4:** Univariate analysis of factors associated with LTFU among HIV-infected adults on antiretroviral therapy

	Univariable Results (*n* = 23890)							
	6 months		12 months		24 months		36 months	
	OR (95% CI)	*P* value	OR (95% CI)	*P* value	OR (95% CI)	*P* value	OR (95% CI)	*P* value
Gender								
Male	1.25 (1.15–1.37)	< 0.001	1.18 (1.09–1.27)	< 0.001	1.13 (1.06–1.21)	< 0.001	1.12 (1.06–1.19)	< 0.001
Female	Ref		Ref		Ref		Ref	
Age groups								
20–35	1.18 (1.08–1.29)	< 0.001	1.31 (1.21–1.41)	< 0.001	1.42 (1.33–1.51)	< 0.001	1.43 (1.34–1.51)	< 0.001
36–50	Ref		Ref		Ref		Ref	
51+	1.00 (0.86–1.16)	0.967	0.97 (0.85–1.10)	0.607	0.95 (0.85–1.07)	0.419	0.97 (0.87–1.08)	0.555
Marital Status								
Single	1.38 (1.22–1.55)	< 0.001	1.34 (1.21–1.48)	< 0.001	1.27 (1.16–1.39)	< 0.001	1.34 (1.24–1.45)	< 0.001
Divorced	1.44 (1.29–1.62)	< 0.001	1.41 (1.28–1.56)	< 0.001	1.4 (1.28–1.52)	< 0.001	1.33 (1.23–1.44)	< 0.001
Married	Ref		Ref		Ref		Ref	
Widowed	0.99 (0.85–1.14)	0.833	0.94 (0.83–1.06)	0.280	0.94 (0.85–1.04)	0.243	0.94 (0.85–1.04)	0.214
BMI								
< 18.5	1.65 (1.41–1.94)	< 0.001	1.48 (1.31–1.67)	< 0.001	1.33 (1.20–1.47)	< 0.001	1.23 (1.13–1.34)	< 0.001
18.5–24.9	Ref		Ref		Ref		Ref	
25–29.9	0.54 (0.41–0.72	< 0.001	0.58 (0.46–0.73)	< 0.001	0.63 (0.53–0.76)	< 0.001	0.66 (0.58–0.76)	< 0.001
30+	0.49 (0.36–0.66)	< 0.001	0.50 (0.41–0.69)	< 0.001	0.61 (0.51–0.73)	< 0.001	0.67 (0.57–0.8)	< 0.001
CD4								
0–250	1.70 (1.47–1.96)	< 0.001	1.6 (1.42–1.80)	< 0.001	1.34 (1.22–1.47)	< 0.001	1.30 (1.20–1.41)	< 0.001
251–500	Ref		Ref		Ref		Ref	
Above 500	1.91 (1.09–3.32)	0.023	1.55 (0.94–2.55)	0.086	1.70 (1.13–2.56)	0.011	1.53 (1.05–2.25)	0.028
Regimen Started								
D4T-based	Ref		Ref		Ref		Ref	
ABC-based	2.09 (1.62–2.68)	< 0.001	2.17 (1.74–2.73)	< 0.001	2.15 (1.74–2.66)	< 0.001	2.01 (1.63–2.48)	< 0.001
AZT-based	0.66 (0.59–0.75)	< 0.001	0.69 (0.62–0.76)	< 0.001	0.73 (0.67–0.80)	< 0.001	0.81 (0.75–0.88)	< 0.001
TDF-based	0.57 (0.51–0.64)	< 0.001	0.62 (0.56–0.68)	< 0.001	0.69 (0.64–0.75)	< 0.001	0.81 (0.75–0.87)	< 0.001
Baseline WHO Stage								
Stage I	Ref		Ref		Ref		Ref	
Stage II	1.16 (0.99–1.37)	0.0707	1.19 (1.04–1.36)	0.01	1.13 (1.01–1.26)	0.032	1.11 (1.02–1.22)	0.0230
Stage III	1.76 (1.51–2.06)	< 0.001	1.63 (1.44–1.84)	< 0.001	1.46 (1.32–1.61)	< 0.001	1.38 (1.26–1.50)	< 0.001
Stage IV	2.45 (1.98–3.04)	< 0.001	1.99 (1.66–2.39)	< 0.001	1.63 (1.40–1.91)	< 0.001	1.53 (1.31–1.78)	< 0.001

**Table 5:** Multivariate analysis of factors associated with LTFU among HIV-infected adults on antiretroviral therapy

	Multivariate Results (*n* = 23890)							
	6 months		12 months		24 months		36 months	
	aOR (95% CI)	*P* value	aOR (95% CI)	*P* value	aOR (95% CI)	*P* value	aOR (95% CI)	*P* value
Gender								
Male	1.23 (1.12–1.36)	< 0.001	1.19 (1.10–1.29)	< 0.001	1.19 (1.1–1.27)	< 0.001	1.19 (1.12–1.27)	< 0.001
Female	Ref		Ref		Ref		Ref	
BMI								
< 18.5	1.52 (1.3–1.78)	< 0.001	2.51 (1.88–3.36)	< 0.001	2.02 (1.63–2.5)	< 0.001	1.68 (1.39–2.02)	< 0.001
18.5–24.9	Ref		Ref		Ref		Ref	
25–29.9	0.58 (0.43–0.77)	< 0.001	0.61 (0.49–0.77)	< 0.001	0.66 (0.55–0.79)	< 0.001	0.69 (0.6–0.79)	< 0.001
30+	0.50 (0.37–0.69)	< 0.001	0.55 (0.42–0.71)	< 0.001	0.62 (0.52–0.75)	< 0.001	0.69 (0.58–0.82)	< 0.001
Age								
20–35	1.23 (1.12–1.35)	< 0.001	1.36 (1.26–1.47)	< 0.001	1.47 (1.37–1.57)	< 0.001	1.49 (1.40–1.59)	< 0.001
36–50	Ref		Ref		Ref		Ref	
51+	1.03 (0.88–1.2)	0.719	1.00 (0.87–1.14)	0.960	0.98 (0.87–1.1)	0.665	0.99 (0.89–1.10)	0.803
Marital Status								
Single	1.34 (1.19–1.51)	< 0.001	1.31 (1.18–1.45)	< 0.001	1.26 (1.15–1.38)	< 0.001	1.34 (1.23–1.45)	< 0.001
Divorced	1.35 (1.19–1.53)	< 0.001	1.31 (1.17–1.46)	< 0.001	1.30 (1.19–1.43)	< 0.001	1.25 (1.15–1.36)	< 0.001
Married	Ref		Ref		Ref		Ref	
Widowed	1.01 (0.87–1.18)	0.859	0.99 (0.87–1.12)	0.814	1.02 (0.91–1.14)	0.726	1.03 (0.93–1.14)	0.555
CD4								
0–250	1.34 (1.16–1.55)	< 0.001	1.33 (1.17–1.5)	< 0.001	1.15 (1.05–1.27)	0.004	1.18 (1.08–1.29)	< 0.001
251–500	Ref		Ref		Ref		Ref	
Above 500	1.42 (0.81–2.49)	0.215	1.22 (0.73–2.05)	0.448	1.42 (0.93–2.16)	0.102	1.33 (0.90–1.96)	0.149
Regimen Started								
D4T-based	Ref		Ref		Ref		Ref	
ABC-based	2.37 (1.82–3.08)	< 0.001	2.44 (1.93–3.09)	< 0.001	2.33 (1.87–2.90)	< 0.001	2.17 (1.75–2.69)	< 0.001
AZT-based	0.74 (0.65–0.83)	< 0.001	0.75 (0.67–0.83)	< 0.001	0.78 (0.71–0.85)	< 0.001	0.85 (0.79–0.93)	< 0.001
TDF-based	0.62 (0.55–0.69)	< 0.001	0.67 (0.61–0.74)	< 0.001	0.73 (0.68–0.8)	< 0.001	0.86 (0.80–0.92)	< 0.001
Baseline WHO Stage								
Stage I	Ref		Ref		Ref		Ref	
Stage II	1.03 (0.87–1.22)	0.732	1.08 (0.95–1.24)	0.242	1.07 (0.96–1.19)	0.251	1.07 (0.98–1.17)	0.147
Stage III	1.34 (1.14–1.57)	< 0.001	1.31 (1.16–1.48)	< 0.001	1.25 (1.13–1.39)	< 0.001	1.22 (1.12–1.34)	< 0.001
Stage IV	1.83 (1.48–2.26)	< 0.001	1.57 (1.31–1.88)	< 0.001	1.38 (1.17–1.62)	< 0.001	1.34 (1.15–1.57)	< 0.001

## Discussion

This analysis included all adults aged 20 years and above initiating ART from 2004 to 2012, who completed 36 months of follow-up. Factors associated with mortality and LTFU outcomes were assessed at different time points during the follow-up period. Despite the improvement in retention during the period under study, the LTFU and mortality increased with each follow-up year, being 14.7% after 1 year of follow-up and increasing to 27.2 and 3% after 1 year and increasing to 4.3% at 36 months respectively.

The factors associated with LTFU and mortality were analyzed at 6, 12, 24, and 36 months. The findings suggest that factors associated with both LTFU and mortality remained constant at all four time-points. LTFU was associated with being male, age 20 to 35 years, single or divorced, advanced WHO clinical stage, low CD4 counts at enrolment, and being on an ABC-based regimen. Mortality risk was higher among men, patients aged 51 years or older, single or divorced, advanced WHO clinical stage, low CD4 cell count at enrolment, and low BMI.

This is similar to data reported elsewhere. A study in the coastal region of Kenya reported that at ART initiation, male gender, older age, and HIV diagnosis at advanced WHO stages were associated with higher rates of attrition, especially within the first year of treatment [10]. A study in China followed up HIV-infected patients on ART for 8 years and reported that younger age, male gender, and being single or divorced was associated with attrition [21]. A study in Zimbabwe also reported that male gender and advanced disease at ART initiation were associated with mortality or LTFU [13].

Despite the higher odds of attrition in men, our study had a proportionately higher number of women. The high number of women in our HIV clinics reflect the national HIV testing patterns which show that a greater proportion of men than women were unaware of their HIV infection [61.9 and 47.8%, respectively] [21]. Men may therefore have presented to HIV clinics with advanced HIV infection. Findings from other studies also suggest that men tend to present with advanced HIV disease at diagnosis and to be at higher risk of LTFU and death [22, 23]. In addition, in SSA, women tend to have better health seeking behavior especially from accessing antenatal care and PMTCT services. HIV testing for men may be improved through male-friendly strategies such as male-friendly clinics, family and partner testing, workplace testing, home testing, and couple testing initiatives [24, 25]. It is also possible that men failed to return to HIV clinics after HIV diagnosis and ART initiation. Targeted interventions such as clinic visit schedules during initial enrolment may improve both retention in care and clinical outcomes for men as recommended in the Kenya HIV treatment guidelines [26]. Less frequent visits thereafter for both clinical assessment and medication pick-ups may improve long term follow-up [11].

Single or divorced status was associated with both LTFU and mortality in our study. A study in the United States found that marital status, especially in men, was associated with mortality from HIV. Divorced and separated individuals were 4.3 times more likely to die of HIV/AIDS than married individuals [27]. Single/never married persons were also 13 times as likely to die of HIV/AIDS compared to their married counterparts [27]. This could be due to single/divorced person’s non-disclosure to sexual partners of their HIV status hence higher likelihood of default, and unstable social relations within the society, resulting in weaker social support systems for the single or divorced patients [28].

The young patients in this study had higher odds of LTFU than older patients. In contrast, older patients had a higher risk of death compared to the young. Young patients tend to be less adherent to ART and to clinic visits [29]. This could be due to stigma and discrimination [30]. These challenges could be addressed through experiential sharing among peers, prompt disclosure, and patient education and counselling [30]. On the other hand, older patients tend to have a higher risk of mortality [31]. A study in China reported that HIV patients with diagnosis at ages between 50 and 59 years were at a 1.60-fold higher risk of death compared to those aged 19–29 years [32]. Older patients are likely to have disease progression despite ART due to a weaker immune system that accompanies old age and more comorbidities. In contrast, younger patients who are retained in HIV programs tend to achieve good viral suppression [33].

In our study, advanced HIV was shown to be associated with above four-fold risk of mortality. This is similar to other studies in SSA which have reported the association of WHO clinical stage III and IV with higher mortality compared to WHO clinical stage I and II [34]. The causes of death are likely due to opportunistic infections and immune reconstitution inflammatory syndrome resulting from severe immunosuppression [35].

Low BMI was found to be associated with increased mortality in our study. Studies in both developed and developing countries have shown that low BMI was associated with mortality [36]. Malnutrition in HIV tends to be due to HIV infection itself but is aggravated by poverty and food insecurity and may be reduced through targeted assessments and correction of malnutrition [37].

Patients with low CD4 between 0 and 250 cells/μl at enrolment were at a higher risk of LTFU in our study. Low median CD4 count at enrolment reflects delays in diagnosis, enrolment into HIV care, and initiation of ART. This data is similar to data published from several parts of SSA [38–41]. Innovative strategies to increase HIV testing are needed followed by same-day linkage and enrolment as well as initiation of ART. Following the adoption of the universal test and treat strategy in Kenya in 2016 [25], there is an opportunity to evaluate the effect of implementing the test and start strategy on LTFU and mortality in Kenya.

Among patients on ABC-containing regimens, there was a two-fold higher odds of mortality at 36 months. While there is evidence of association between ABC use and risk of myocardial infarction, cardiovascular risk assessment was not included in this observational cohorts [42]. It is also notable that since 2010, Kenya adopted the use of TDF-containing regimens as the preferred first-line ART for adults to reduce the likelihood of toxicities related to the use of non-TDF containing regimens [3].

This data set was large making it possible to infer results. However, we had several limitations particularly because of missing data from the electronic database. This issue was addressed via multiple imputation during the regression analysis. Also, some patients that were recorded as LTFU were probably enrolled in other facilities following decentralization initiatives which began in 2008, resulting in higher attrition rates for our study [43]. In addition, the use of unique patient identifiers is yet to be adopted in Kenya, making inter-facility tracing of patients challenging. Finally, gaps in documentation could have resulted in misclassification of dead patients as LTFU.

## Conclusions

Scale up of ART has been successful but attrition remains a challenge in Kenya. Factors associated with LTFU and mortality were generally similar over a 36-month follow-up period. Implementation of HIV treatment programs should therefore be tailored based on gender, age profiles, nutritional, and, marital status of patients. Interventions targeting retention in treatment should be tailored to the needs of younger patients. It was notable that men had a higher risk of both LTFU and mortality outcomes. In addition, programs should focus on the care of older patients to reduce the risk of mortality. At the time of preparing this manuscript, it was noted that Kenya had already adopted differentiated approaches to care in 2016, presenting an opportunity for tailored approaches to the care of patients based clinical characteristics, sub-population, and context.

## Data Availability

The data used for this analysis is a deidentified dataset of individual level routine HIV care and treatment data and is not currently publically available as it is property of Ministry of Health and Government of Kenya. However, the dataset can be obtained from the corresponding author based on a reasonable request.

## Abbreviations

ABC: Abacavir
ART: Antiretroviral therapy
AZT: Zidovudine
BMI: Body Mass Index
CDC: Centers for Disease Control and Prevention
d4T: Stavudine
LTFU: Loss to follow-up
NASCOP: National AIDS and STI Control Program
NCD: Noncommunicable disease
PEPFAR: President’s Emergency Plan for AIDS Relief
PITC: Provider initiated testing and counselling
PLHIV: People living with HIV
PMTCT: Prevention of mother-to-child transmission of HIV
SSA: Sub-Saharan Africa
TDF: Tenofovir
WHO: World Health Organization
